# Distinct Patterns of GR Transcriptional Regulation in Liver and Muscle of LPS-Challenged Weaning Piglets

**DOI:** 10.3390/ijms23158072

**Published:** 2022-07-22

**Authors:** Jie Liu, Yidan Jiang, Zheng Jiang, Yue Feng, Ruqian Zhao

**Affiliations:** 1 MOE Joint International Research Laboratory of Animal Health & Food Safety, Nanjing Agricultural University, Nanjing 210095, China; 2019207040@njau.edu.cn (J.L.); 2020107033@stu.njau.edu.cn (Y.J.); ljzs20121229@163.com (Z.J.); 2016107034@njau.edu.cn (Y.F.); 2 Key Laboratory of Animal Physiology & Biochemistry, Nanjing Agricultural University, Nanjing 210095, China

**Keywords:** pig, liver, muscle, GR, transcriptional regulation, tissue specificity

## Abstract

Glucocorticoid receptor (GR), which is ubiquitously expressed in nearly all cell types of various organs, mediates the tissue-specific metabolic and immune responses to maintain homeostasis and ensure survival under stressful conditions or pathological challenges. The neonatal period is metabolically demanding, and piglets are subjected to multiple stressors in modern intensive farms, especially around weaning. The liver is more responsive to LPS challenge compared to muscle, which is indicated by significantly increased TLR4 and p-p65, TNF-α, and IL-6 levels in association with GR down-regulation at both mRNA and protein levels. GR binding to the putative nGRE on TNF-α and IL-6 gene promoters decreased in the liver, but not muscle, upon LPS stimulation. The transcriptional regulation of GR also showed striking differences between liver and muscle. GR exon 1 mRNA variants 1–4, 1–5, and 1–6 were down-regulated in both liver and muscle, but a significant up-regulation of GR exon 1–9/10 mRNA variants abolished the change of total GR mRNA in the muscle in response to LPS stimulation. The significant down-regulation of GR in the liver corresponded with significantly decreased binding of p-GR and diminished histone acetylation in GR gene promoters. These results indicate that tissue-specific GR transcriptional regulation is involved in the differential inflammation responses between liver and muscle.

## 1. Introduction

In modern intensive farms, pigs are often exposed to various stressors that may induce chronic systemic inflammation. The occurrence of inflammatory response is related to the activation of the hypothalamic–pituitary–adrenal axis (HPA) axis, which in turn releases cortisol to coordinate the body’s defensive and metabolic systems for survival. Cortisol acts predominantly through binding to the glucocorticoid receptor (GR) [1], which is ubiquitously expressed in nearly all cell types of various organs and mediates the tissue-specific metabolic and immune responses to stressful conditions or pathological challenges [2]. GR deficiency, specifically in macrophages, causes exaggerated immune responses in mice, including infiltration of neutrophils, T cells, and macrophages in the colon, associated with enhanced serum IL-6 level [3]. Microglial-specific GR knockout leads to more severe cellular damage, neuronal degeneration, and higher pro-inflammatory cytokines release in response to lipopolysaccharide (LPS) challenge [4]. GR is underlying the action of many anti-inflammatory and immunosuppressive drugs that are widely used in human medicine, yet the specificity and sensitivity have been a concern in the development of novel GR agonists with harmful side effects [5].

The neonatal period is metabolically demanding, and piglets are subjected to multiple stressors, including pathogenic microbes in modern intensive farms, especially around weaning [6,7]. LPS, the major component of the outer membrane of Gram-negative bacteria [8], is known to be a key pathogen-associated molecular pattern (PAMP). LPS can stimulate the innate immune system via the innate pattern recognition receptor Toll-like receptor 4 (TLR4) [9], which mediates a local or systemic inflammatory response. LPS can also stimulate non-immune cells (such as cardiomyocytes) and initiate the inflammatory process [10]. However, it remains unknown whether LPS-induced inflammatory responses show tissue specificity in pigs.

It has been reported that LPS causes the activation of the HPA axis, which promotes the secretion of glucocorticoids (GCs) by adrenal glands to reduce inflammatory response [11,12]. The tissue-specific immune responses are closely related to the tissue specificity and sensitivity of GR. GR expression is predominantly regulated at the level of transcription. GR protein content is positively correlated with its mRNA level in LPS-injected pigs [13]. Many transcription factors, such as SP1, CREB, YY1, and Ets1, are identified to activate or inhibit GR transcription through binding to the GR alternative promoters [14,15,16,17]. In addition, GR can act as its own transcription factor to regulate its transcription [18,19]. Muscle and liver are the most important organs in the regulation of immune and metabolic homeostasis. Our previous research indicates that the total GR and its exon 1 mRNA variants are expressed in a tissue-specific manner in the muscle and liver of pigs, which contributes to the tissue-specific expression of GR protein [20]. However, it is unclear whether GR is subjected to tissue-specific regulation and how this is related to the distinct LPS-induced inflammatory responses in the liver and muscle of weaning piglets.

Therefore, the present study aimed to demonstrate the tissue-specific inflammatory responses to LPS challenge and its relationship with tissue-specific expression of GR in the liver and muscle of weaning piglets.

## 2. Results

### 2.1. Distinct Inflammatory Responses Caused by LPS Challenge in Liver and Muscle

**Figure 1:**
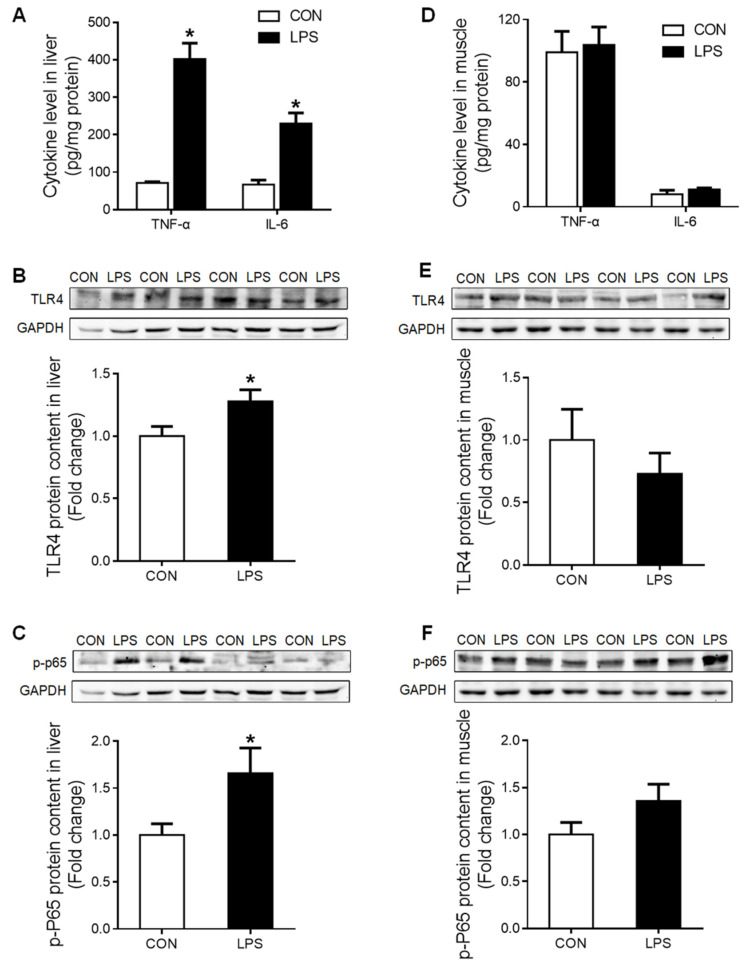
Inflammatory response in liver and muscle when treatment with LPS. (**A**) Liver cytokine levels of TNF-α and IL-6. (**B**) Western blot and densitometric analyses of TLR4 and GAPDH proteins in the liver. (**C**) Western blot and densitometric analyses of p-P65 and GAPDH proteins in the liver. (**D**) Muscle cytokine level of TNF-α and IL-6. (**E**) Western blot and densitometric analyses of TLR4 and GAPDH proteins in muscle. (**F**) Western blot and densitometric analyses of p-P65 and GAPDH proteins in muscle. Values were expressed as means ± SEM, *n* = 4 in each group, * *p* < 0.05.

The pro-inflammatory cytokines tumor necrosis factor α (TNF-α) and interleukin-6 (IL-6) were significantly increased (*p* < 0.05) in the liver of LPS-challenged piglets (Figure 1A), which was associated with significantly up-regulated (*p* < 0.05) TLR-4 (Figure 1B) and p-p65 NF-κB (Figure 1C) proteins. In contrast, no responses to LPS challenge were detected in any of these parameters in the muscle (Figure 1D–F).

### 2.2. Cortisol Content and GR Expression in Liver and Muscle in Response to LPS Challenge

**Figure 2:**
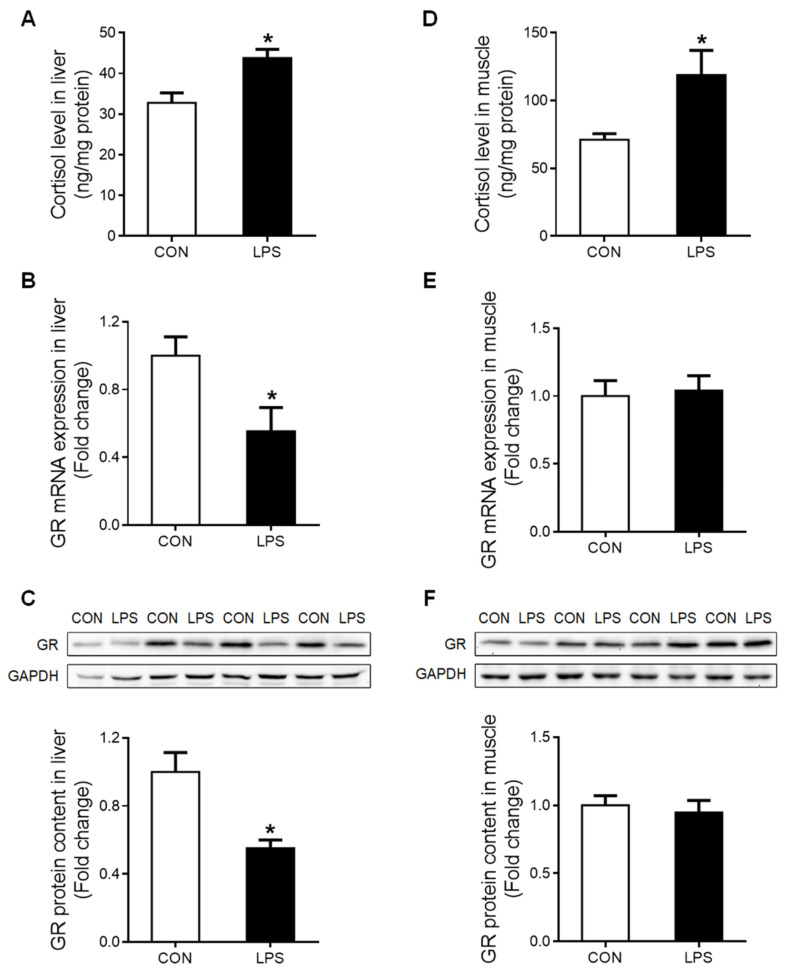
Cortisol content and GR expression in liver and muscle in response to LPS challenge. (**A**) Liver cortisol level. (**B**) GR mRNA expression of the liver was evaluated by qRT-PCR. (**C**) Western blot and densitometric analyses of GR and GAPDH proteins in the liver. (**D**) Muscle cortisol level. (**E**) GR mRNA expression of muscle was evaluated by qRT-PCR. (**F**) Western blot and densitometric analyses of GR and GAPDH proteins in muscle. Values were expressed as means ± SEM, *n* = 4 in each group, * *p* < 0.05.

The tissue content of cortisol was significantly increased (*p* < 0.05) in both liver (Figure 2A) and muscle (Figure 2D) under LPS treatment. However, a significant down-regulation (*p* < 0.05) of total GR at both mRNA (Figure 2B) and protein (Figure 2C) levels was detected in the liver but not in the muscle (Figure 2E,F).

**Figure 3:**
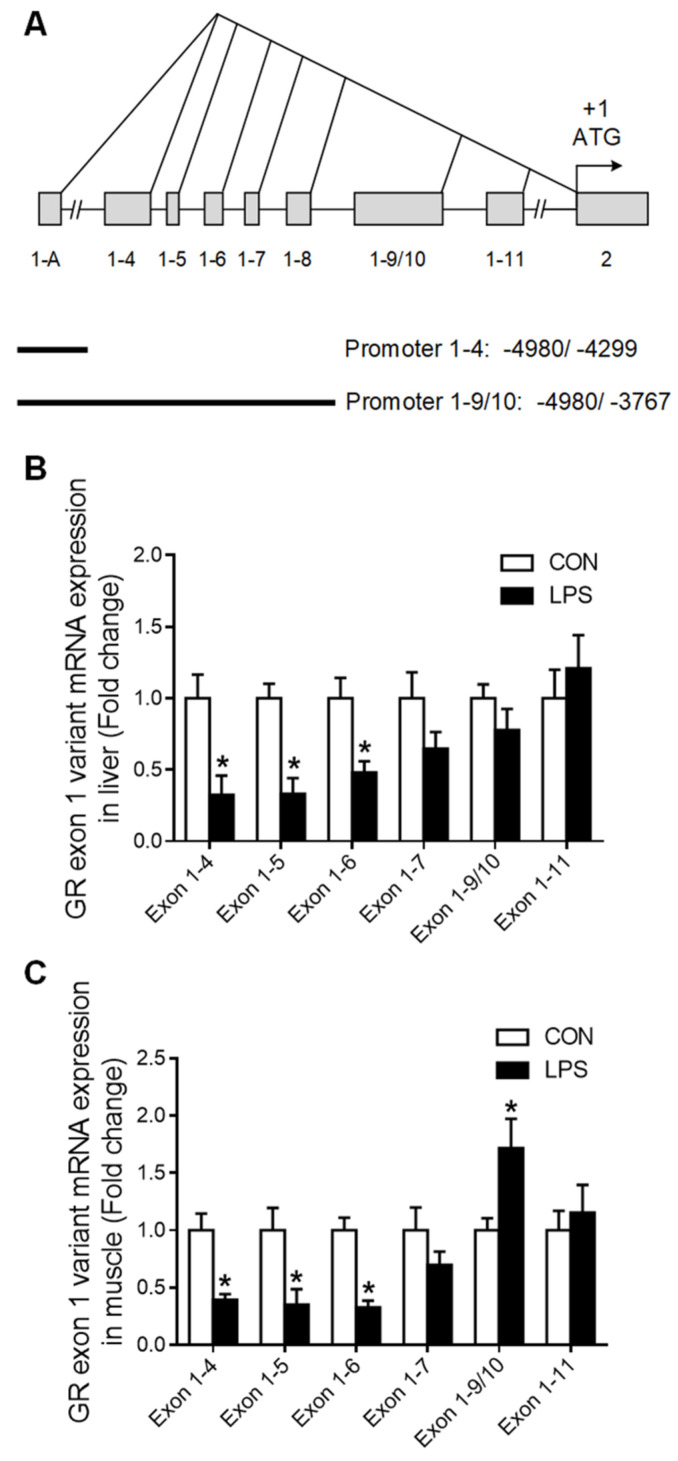
GR exon 1 variants expression in liver and muscle when treatment with LPS. (**A**) Genomic location of the porcine GR alternative promoters. (**B**) GR exon 1 variants expression in liver. (**C**) GR exon 1 variants expression in muscle. Values were expressed as means ± SEM, *n* = 4 in each group, * *p* < 0.05.

The 5′-heterogeneity of porcine GR exon 1 mRNA variants (Figure 3A) was further investigated. The expression of GR exon 1 mRNA variants 1–4, 1–5, and 1–6 was significantly reduced (*p* < 0.05) in both liver (Figure 3B) and muscle (Figure 3C) in response to LPS challenge. However, GR exon 1–9/10 mRNA showed a tissue-specific response to LPS, which was unchanged in the liver (Figure 3B) but significantly up-regulated (*p* < 0.05) in the muscle (Figure 3C).

### 2.3. Transcriptional Regulation of GR in Liver and Muscle under LPS Treatment

**Figure 4:**
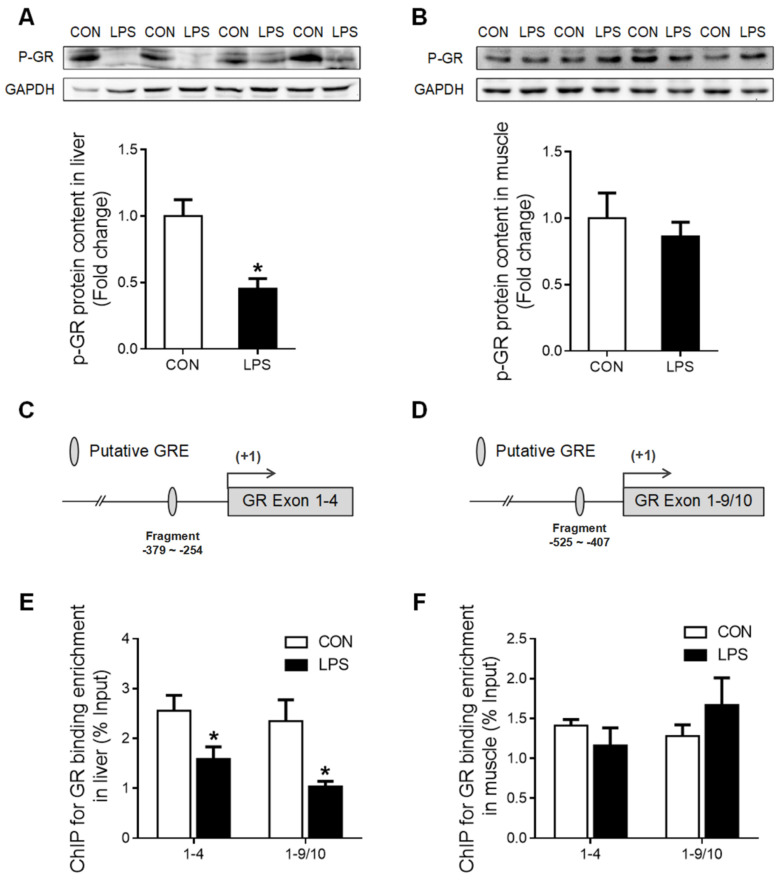
The change of p-GR and GR binding with its promoter in liver and muscle under LPS treatment. (**A**) Western blot and densitometric analyses of p-GR and GAPDH proteins in the liver. (**B**) Western blot and densitometric analyses of p-GR and GAPDH proteins in muscle. (**C**) The putative fragment GRE site on the promoter region of GR exon 1–4. (**D**) The putative fragment GRE site on the promoter region of GR exon 1–9/10. (**E**) ChIP analyses GR binding to GRE sites on the promoter of GR exon 1–4 and 1–9/10 in the liver. (**F**) ChIP analyses p-GR binding to GRE sites on the promoter of GR exon 1–4 and 1–9/10 in muscle. Values were expressed as means ± SEM, *n* = 4 in each group, * *p* < 0.05.

Western blot analysis showed that phosphorylated GR (p-GR) was significantly decreased (*p* < 0.05) in the liver (Figure 4A) but unchanged in the muscle (Figure 4B). A putative glucocorticoid response element (GRE) was predicted on the promoter region of both GR exon 1–4 (Figure 4C) and 1–9/10 (Figure 4D). ChIP analysis showed that GR binding with GR promoter 1–4 and 1–9/10 was significantly down-regulated (*p* < 0.05) in LPS-treated porcine liver (Figure 4E), but no alterations were detected in the muscle (Figure 4F).

**Figure 5:**
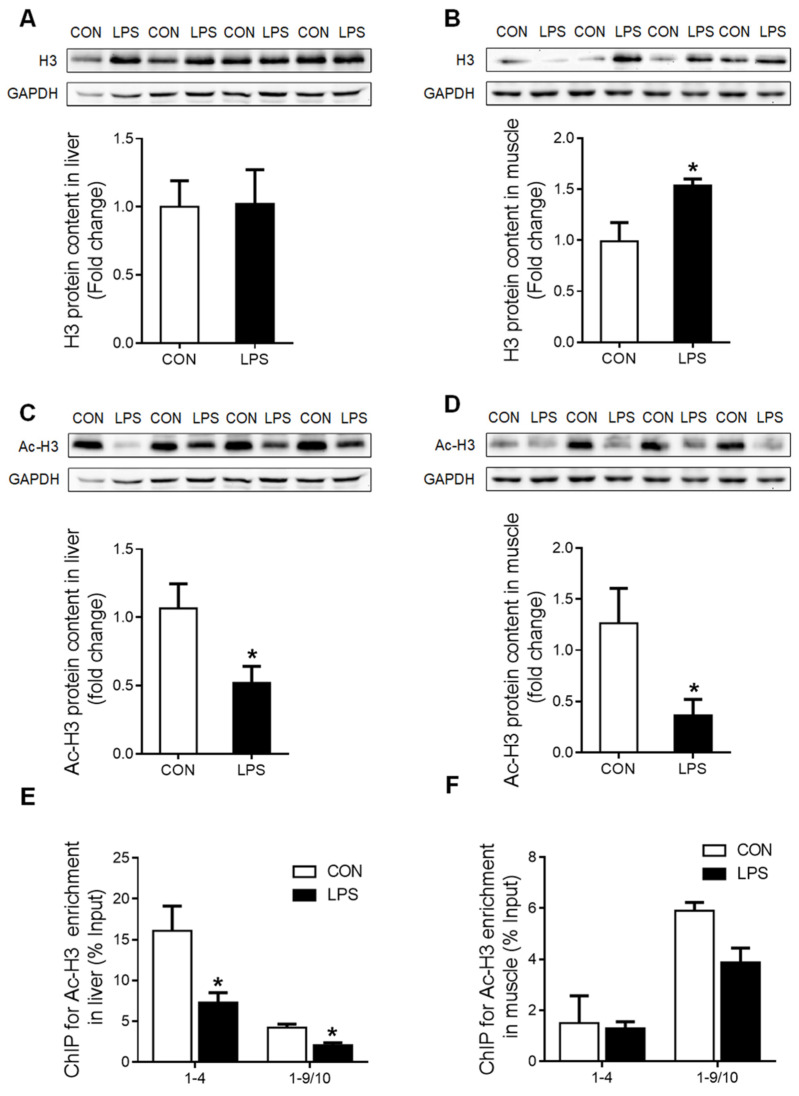
Histone H3 protein content and the enrichment of histone H3 acetylation on GR promoters 1–4 and 1–9/10 under LPS stimulation. (**A**) Western blot and densitometric analyses of H3 and GAPDH proteins in the liver. (**B**) Western blot and densitometric analyses of H3 and GAPDH proteins in muscle. (**C**) Western blot and densitometric analyses of the global histone H3 acetylation level in the liver. (**D**) Western blot and densitometric analyses of the global histone H3 acetylation level in muscle. (**E**) ChIP analyses for Ac-H3 enrichment on the promoter of GR exon 1–4 and 1–9/10 in the liver. (**F**) ChIP analyses Ac-H3 enrichment on the promoter of GR exon 1–4 and 1–9/10 in muscle. Values were expressed as means ± SEM, *n* = 4 in each group, * *p* < 0.05.

Moreover, histone H3 protein content was unchanged in the liver (Figure 5A) but significantly up-regulated (*p* < 0.05) in the muscle (Figure 5B) of LPS-treated piglets. Nevertheless, the global histone H3 acetylation level was significantly reduced (*p* < 0.05) both in the liver (Figure 5C) and the muscle (Figure 5D) upon LPS challenge. ChIP assay showed that the enrichment of histone H3 acetylation on GR promoters 1–4 and 1–9/10 was significantly decreased (*p* < 0.05) in the liver of LPS-treated piglets (Figure 5E), while no change was detected in the muscle (Figure 5F).

### 2.4. GR Binding to TNF-α and IL-6 Gene Promoters in Liver and Muscle

**Figure 6:**
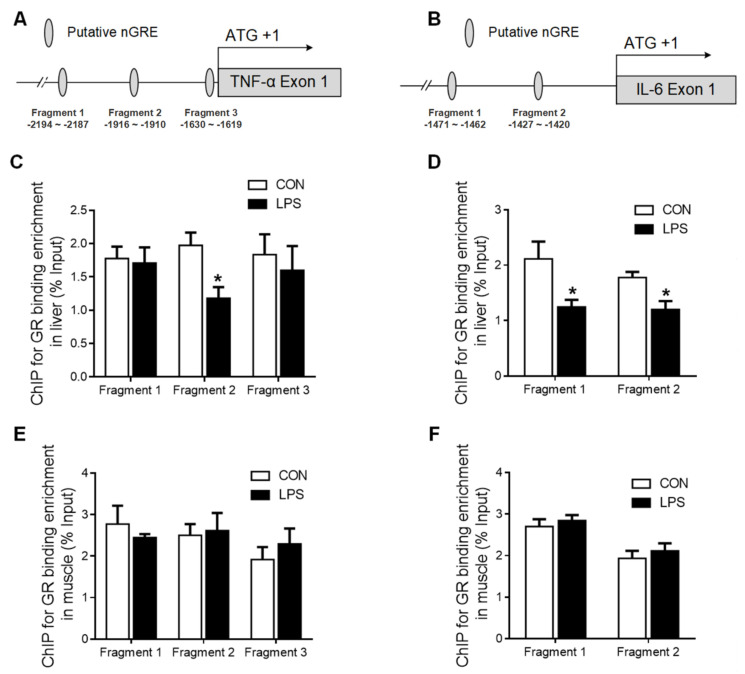
GR binding to TNF-α and IL-6 gene promoters in liver and muscle. (**A**) The putative fragment nGRE site on the promoter region of TNF-α. (**B**) The putative fragment nGRE site on the promoter region of IL-6. (**C**) ChIP analyses GR binding to nGRE sites on the promoter of TNF-α exon 1–4 and 1–9/10 in the liver. (**D**) ChIP analyses GR binding to nGRE sites on the promoter of IL-6 exon 1–4 and 1–9/10 in the liver. (**E**) ChIP analyses GR binding to nGRE sites on the promoter of TNF-α exon 1–4 and 1–9/10 in muscle. (**F**) ChIP analyses GR binding to nGRE sites on the promoter of IL-6 exon 1–4 and 1–9/10 in muscle. Values were expressed as means ± SEM, *n* = 4 in each group, * *p* < 0.05.

Several putative negative glucocorticoid response elements (nGRE) were predicted on promoter regions of TNF-α (Figure 6A) and IL-6 (Figure 6B) genes using the bioinformatics method (www.gene-regulation.com). The binding of GR to these putative nGREs in liver and muscle tissues was validated by using ChIP-qPCR. GR binding to fragment 2 of the TNF-α (Figure 6C) gene promoter, as well as to both fragment 1 and 2 of the IL-6 gene promoter, was significantly decreased (*p* < 0.05) in the liver upon LPS challenge (Figure 6D). However, no changes in GR binding to TNF-α or IL-6 gene promoters were detected in the muscle in response to LPS challenge (Figure 6E,F).

## 3. Discussion

In the present study, we provide evidence that tissue-specific GR expression and regulation contribute to distinct inflammatory responses to LPS challenge in the liver and the muscle of piglets. The liver displays a more significant inflammatory response to LPS challenge compared to muscle, which is indicated by significantly activated TLR4/p-p65 pathway and elevated serum TNF-α andIL-6 levels, in association with GR down-regulation at both mRNA and protein levels. In contrast, muscle did not show inflammatory responses to LPS challenge, which coincided with a distinct pattern of GR expression and regulation.

Tissue-specific responses of pro-inflammatory mediators to LPS attribute to various factors, including the cell type composition, the blood flow rate, and LPS bioavailability, as well as LPS signaling and its regulatory mechanisms in the tissue. Previously, we reported distinct responses of LPS-induced inflammatory responses in the liver and the hypothalamus of chickens [21]. The liver is directly exposed to LPS and Kupffer cells, and its resident macrophages initiate the inflammatory responses by releasing pro-inflammatory cytokines such as TNF-α and IL-6, which communicate with the hepatocytes to induce the acute phase responses [22]. However, the hypothalamus is protected by the blood–brain barrier (BBB) from direct exposure to cytokines or LPS in the peripheral circulation. Muscle inflammation requires higher concentrations of LPS than the liver. A low concentration of LPS (2.5 mg/kg) can induce liver injury and inflammation [23,24,25], but high-dose of LPS (12 mg/kg) stimulates cytokine expression in mouse skeletal muscle [26]. On the other hand, the tissue specificity of cortisol action, which is mediated by GR, may also play a role in the tissue-specific LPS response.GR is a target of many potent anti-inflammatory drugs [27]. It is known that many of these drugs have tissue-specific effects [28]. For instance, dexamethasone can relieve LPS-induced inflammation in peripheral tissues but not in the brain in mice [29]. Therefore, tissue-specific distribution of GR may contribute to distinct inflammatory responses to LPS in the liver and muscle of piglets.

Abundant studies have pointed out that GR is transcribed in a tissue- and cell type-specific manner with multiple exon 1 mRNA variants driven by selective promoters [30,31,32,33]. At least 11 splice variants in the 5′-UTR region of exon 1 of the human GR gene have been identified. Each exon 1 mRNA variant has its individual proximal promoter region that is involved in the control of tissue-specific GR expression [33]. In pigs, GR exon 1–9/10 is identified as the most abundant GR transcript in the liver, comprising 50% of total GR mRNA. While in the muscle, GR exon 1–4 was the highest, accounting for 32% of the total GR mRNA [20]. The tissue-specific transcription of GR exon 1 variants is regulated by selective promoter usage in different tissues [34]. The promoter activity analyses found that the promoter for porcine GR exon 1–9/10 demonstrated the strongest trans-activity, which explains the fact that GR is most abundantly expressed in the porcine liver [20]. Moreover, GR expression in response to cortisol is also tissue-specific. For example, T lymphocytes increased their GR expression in response to corticosteroids [35,36], while the opposite response was reported in the hippocampus [37]. A similar phenomenon was observed in the present study, in which [36] GR exon 1 mRNA variants 1–4, 1–5, and 1–6 were down-regulated in both liver and muscle in response to LPS, but GR exon 1–9/10 mRNA variant was significantly up-regulated only in the muscle. The increase in GR exon 1–9/10 may counteract the decrease in exons 1–4, 1–5, and 1–6, leading to unchanged overall GR mRNA and protein in the muscle of LPS-treated piglets.

Overall, GR expression can be autoregulated by glucocorticoids such as cortisol. Multiple GREs and negative GREs have been identified in the promoter region of GR [38]. GREs are predicted on the promoters of GR exons 1–4 and 1–9/10 that demonstrated significantly higher activities than other promoters in porcine liver and muscle [20]. In the present study, the down-regulation of GR expression in the liver coincided with decreased GR activation, indicated by GR phosphorylation, and the binding of endogenous GR to the GREs on the promoter of GR exon 1–4 and 1–9/10. While in the muscle, neither overall GR expression/phosphorylation nor GR binding to the GREs of the promoters were changed upon LPS challenge. Obviously, other transcription factors and post-translational modifiers involved in GR regulation cannot be excluded in the tissue-specific expression and regulation of GR. Phosphorylation of GR can affect its ligand- and DNA-binding affinity, subcellular trafficking, and cofactor recruitment [39,40,41], consequently leading to a modified anti-inflammatory potential [42].

The tissue-specific regulation of GR is also related to the specific distribution of histone acetylation on GR promoters. Histone deacetylase 6 (HDAC6) can be recruited by GR to reduce the histone acetylation level on GR promoter fragment −1523/−8, thereby inhibiting its transcription under dexamethasone treatment [43]. LPS has been reported to regulate the immune and inflammatory responses by reducing the level of histone acetylation [44], and suberoylanilide hydroxamic acid, an HDAC inhibitor, can attenuate the inflammatory response [45].

Although GR is expressed in virtually all types of mammalian cells, it regulates the expression of distinct sets of genes in a promoter- and cell type-specific manner [46,47]. Even in immune cells, the physiological outcomes of GR activation are highly cell type-specific. For example, glucocorticoids are anti-apoptotic in neutrophils but pro-apoptotic in eosinophils, dendritic, and some T cells [48]. The determinants of this promoter selectivity are not completely understood. GR binds GREs directly and typically activates target genes under LPS treatment, including anti-inflammatory mediators such as Dusp1 and Gilz [49,50]. Equally common are nGREs, where GR associates with other DNA-bound regulators, most prominently NF-κB or AP1, and represses their pro-inflammatory target genes, including TNF-α and IL-6 [51]. We found that nGRE exists in the promoters of TNF-α and IL-6, and GR may directly inhibit their transcription in the liver but not in the muscle.

**Figure 7:**
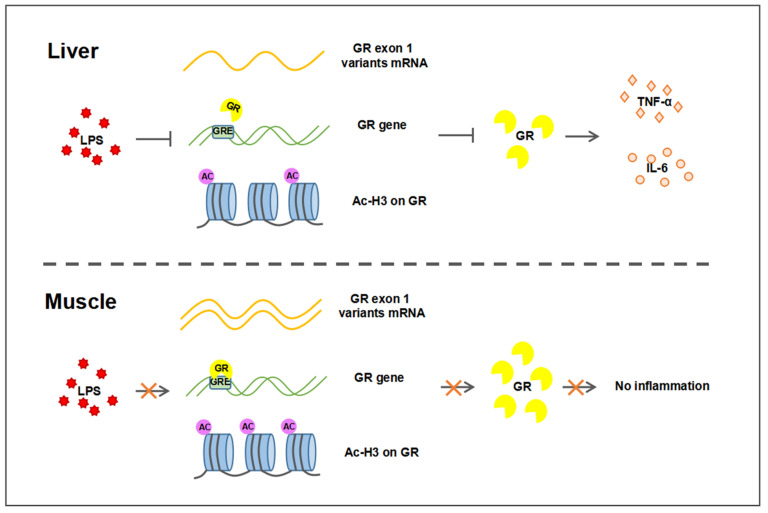
Different transcriptional regulation of GR in liver and muscle after LPS challenge. The decrease in GR expression in the liver after LPS stimulation was associated with the decrease in GR exon 1–4/5/6 expression, the binding of GR to GRE, and the acetylation of H3. Decreased GR results in increased expression of inflammatory factors TNF-α and IL-6, leading to an inflammatory response. In muscle, LPS did not affect the expression of GR, so there was no inflammatory response. Arrows for promotion, lines for inhibition, and crosses for no influence.

In summary, the present study demonstrated that GR transcription was down-regulated upon LPS challenge in the porcine liver, but not muscle, which involved tissue-specific GR autoregulation and histone acetylation on its promoters. The regulation of TNF-α and IL-6 expression by tissue-specific transcription-regulated GR leads to LPS-induced tissue-specific inflammatory responses, as shown in Figure 7. Our findings provide insight into the role of GR in regulating tissue-specific inflammatory responses.

## 4. Materials and Methods

### 4.1. Ethics Statement

The experimental protocol was approved by the Animal Ethics Committee of Nanjing Agricultural University, with project number 2016YFD0500502. The sampling procedures complied with the “Guidelines on Ethical Treatment of Experimental Animals” (2006) no. 398 set by the Ministry of Science and Technology, China.

### 4.2. Animals and Treatment

A total of 12 hybrid (Landrace × Large White × Duroc) male piglets were obtained from a pig breeding farm in Changzhou, Jiangsu Province, China, immediately after weaning. After one week of acclimatization, the piglets were randomly assigned to the LPS group (15 µg/kg, i.m., *n* = 6) and the control group (same volume of saline, i.m., *n* = 6). Six hours after injection, the piglets were killed for sampling. Blood samples were collected immediately, and the serum samples were stored at −20 °C. Liver (no gall bladder) and muscle samples were taken within 20 min postmortem, then snap-frozen in liquid nitrogen and stored at −80 °C for further analysis.

### 4.3. Liver and Muscle Cortisol Assay

Liver and muscle samples (200 mg) were homogenized in 1 mL RIPA buffer solution (50 mM Tris-HCl pH 7.5, 150 mM NaCl, 1% Triton X-100, 0.1% SDS) and centrifuged at 12,000× *g* rpm for 20 min at 4 °C. A total of 3 mL of dichloromethane were added to 300 μL supernatant. The mixture was centrifuged at 8000× *g* rpm for 15 min at room temperature, and the upper aqueous phase was discarded. The dichloromethane phase was aspirated into a new centrifuge tube, dried in a fume hood overnight, and reconstituted with 100 μL of water. The cortisol content of each sample was determined by using Iodine (125I) Cortisol Radioimmunoassay Kit (KIPI28000, Beijing North Institute of Bio Co., Ltd., Beijing, China) according to the manufacturer’s instructions.

### 4.4. Determination of Liver and Muscle Concentrations of IL-6 and TNF-α

Briefly, liver and muscle samples (40 mg) were homogenized in RIPA buffer solution and centrifuged at 12,000× *g* rpm for 20 min at 4 °C. Supernatants were used in the determination of TNF-α and IL-6 with Quantikine^®^ ELISA Porcine TNF-α Immunoassay kit (PTA00, R&D, Minneapolis, MN, USA), and Quantikine^®^ ELISA Porcine IL-6 Immunoassay kit (P6000B, R&D, Minneapolis, MN, USA), respectively.

### 4.5. Total RNA Isolation and Real-Time PCR

Total RNA was isolated from liver and muscle samples using TRIzol Reagent (Invitrogen, Waltham, MA, USA) and reverse-transcribed to cDNA using a first strand cDNA synthesis kit (Promega, Madison, WI, USA). The cDNA was diluted 20-fold for real-time PCR with an Mx3000 P Real-Time PCR System (Stratagene, La Jolla, CA, USA). All primers (Table 1) were synthesized by Genscript Biotech (Nanjing, China). Several reference genes were tested, and β-actin was chosen as a reference gene. Data were analyzed using the method of 2^−ΔΔCT^.

### 4.6. Total Cytosolic Protein Extraction and Western Blotting

Total protein was extracted from 40 mg of frozen liver and muscle samples as previously described [52]. The protein concentrations were measured with a BCA Protein Assay kit (No.23225, Thermo Scientific, Waltham, MA, USA) according to the manufacturer’s instructions. SDS-PAGE gel electrophoresis was performed with 20 μg of protein and then transferred to a nitrocellulose membrane. The membranes were blocked with 3% skim milk and incubated with antibodies. TLR4 (SC-1074, Santa Cruz Biotechnology, Dallas, TX, USA), GR (SC-1004, Santa Cruz Biotechnology, Dallas, TX, USA), and p-p65 NF-κB (S536, Bioworld, Nanjing, China) antibodies were diluted according to the instructions. The GAPDH (AP0060, Bioworld, Nanjing, China, diluted 1:5000) was selected as an internal control. Western blot results were analyzed using Quantity One software 4.6.6 (Bio-Rad, Hercules, CA, USA).

### 4.7. Chromatin Immunoprecipitation (ChIP) Assay

ChIP analysis is the same as our previous method [53]. A total of 200 mg of ground tissue was washed with PBS containing a protease inhibitor (11697498001, Roche, Basel, Switzerland). The reaction was crosslinked with 1% formaldehyde and terminated with 2.5 mol/L of glycine. After washing with PBS, it was lysed with SDS lysis buffer containing protease inhibitor. Chromatin was ultrasonized to an average length of ~500 bp and diluted in ChIP dilution buffer precleared with salmon sperm DNA/protein G agarose beads (60 mL, 50% slurry). The precleared chromatin preparations were incubated with 2 mg of primary antibody overnight at 4 °C. Antibody against GR (M20, sc-1004x, Santa Cruz, Dallas, TX, USA) and Ac-H3 (ab4729, Abcam, Cambridge, UK) were used. A negative control was included with normal rat IgG, or no antibody was added. Protein G agarose beads (120 mL, 50% slurry) were added to capture the immunoprecipitated chromatin complexes. Finally, reverse cross-linking was performed to release DNA fragments from the immunoprecipitated complex at 65 °C for 5 h, and the DNA was purified. Immunoprecipitated DNA was used as a template for real-time PCR using specific primers to amplify genomic sequences at the promoter regions of TNF-α, IL-6, and GR genes (Table 1).

### 4.8. Statistical Analysis

All data are presented as means ± SEM, and the differences between groups were analyzed using an independent-samples *t*-test with SPSS 20.0 software (SPSS Inc., Chicago, IL, USA). The differences were considered statistically significant when *p* < 0.05.

## Figures and Tables

**Table 1:** Nucleotide sequences of primers.

Target Genes	Primer Sequences (5′ to 3′)		Used For
GR exon1–4	F: ATCGTAATATGTGCGGCC	R: TTGCTTCCTGAGCCTTTT	Real-time PCR
GR exon1–5	F: AACTTGGATGCGGGCCC	R: TTGCTTCCTGAGCCTTTT	Real-time PCR
GR exon1–6	F: ATGCGGGGGAGGGGGACC	R: ACGCTGCTGGGGATTTC	Real-time PCR
GR exon1–7	F: GCGGCGAAGAGAAACTAGAG	R: ACGCTGCTGGGGATTTC	Real-time PCR
GR exon1–9/10	F: CCTGCTTTCACACGCTAA	R: ACGCTGCTGGGGATTTC	Real-time PCR
GR exon1–11	F: AACTTGGATGCGGGCC	R: TTGCTTCCTGAGCCTTTT	Real-time PCR
Total GR	F: CCAAGGAATCGCTGACCCAG	R: ATTGCTTCCTGAGCCTTTTGG	Real-time PCR
TNF-α Fragment 1	F: CGCTCAGAAGTCCGTGTGC	R: GTCATAAGGGAGGTGAAAAGGG	ChIP PCR
TNF-α Fragment 2	F: GCCTTCTCCCCTTTTCACCT	R: CAGATCCTCAGCAGAGCATCC	ChIP PCR
TNF-α Fragment 3	F: TGAGGGACTTTGAACGGATGA	R: CAGATACGAGCAGACAGCATTTC	ChIP PCR
IL-6 Fragment 1	F: TTGTTCAGTGCTGGAGGGTTG	R: TGCTTGGGTTTGCTTCGC	ChIP PCR
IL-6 Fragment 2	F: CAAACCCAAGCAGATTGTGATG	R: CAGTTGCCCAGACACCCG	ChIP PCR
GR exon1–4 Fragment	F: AGACTTAGGTGTGATTCTGCGGA	R: CATTGATATGACTGACGGTGGC	ChIP PCR
GR exon1–9/10 Fragment	F: CTGAGATACCGCCATCCTAAAGT	R: GGGCTCTGTCCACAAACCATT	ChIP PCR

## Data Availability

Not applicable.
